# Gender difference in symptomatic radiographic knee osteoarthritis in the Knee Clinical Assessment – CAS(K): A prospective study in the general population

**DOI:** 10.1186/1471-2474-9-82

**Published:** 2008-06-11

**Authors:** Rosie J Lacey, Elaine Thomas, Rachel C Duncan, George Peat

**Affiliations:** 1 Primary Care Musculoskeletal Research Centre, Keele University, Keele, Staffordshire ST5 5BG, UK

## Abstract

**Background:**

A recent study of adults aged ≥50 years reporting knee pain found an excess of radiographic knee osteoarthritis (knee ROA) in symptomatic males compared to females. This was independent of age, BMI and other clinical signs and symptoms. Since this finding contradicts many previous studies, our objective was to explore four possible explanations for this gender difference: X-ray views, selection, occupation and non-articular conditions.

**Methods:**

A community-based prospective study. 819 adults aged ≥50 years reporting knee pain in the previous 12 months were recruited by postal questionnaires to a research clinic involving plain radiography (weight-bearing posteroanterior semiflexed, supine skyline and lateral views), clinical interview and physical examination. Any knee ROA, ROA severity, tibiofemoral joint osteoarthritis (TJOA) and patellofemoral joint osteoarthritis (PJOA) were defined using all three radiographic views. Occupational class was derived from current or last job title. Proportions of each gender with symptomatic knee ROA were expressed as percentages, stratified by age; differences between genders were expressed as percentage differences with 95% confidence intervals.

**Results:**

745 symptomatic participants were eligible and had complete X-ray data. Males had a higher occurrence (77%) of any knee ROA than females (61%). In 50–64 year olds, the excess in men was mild knee OA (particularly PJOA); in ≥65 year olds, the excess was both mild and moderate/severe knee OA (particularly combined TJOA/PJOA). This male excess persisted when using the posteroanterior view only (64% vs. 52%). The lowest level of participation in the clinic was symptomatic females aged 65+. Within each occupational class there were more males with symptomatic knee ROA than females. In those aged 50–64 years, non-articular conditions were equally common in both genders although, in those aged 65+, they occurred more frequently in symptomatic females (41%) than males (31%).

**Conclusion:**

The excess of knee ROA among symptomatic males in this study seems unlikely to be attributable to the use of comprehensive X-ray views. Although prior occupational exposures and the presence of non-articular conditions cannot be fully excluded, selective non-participation bias seems the most likely explanation. This has implications for future study design.

## Background

Gender differences in the occurrence of knee osteoarthritis are well documented. Population studies in developed and developing countries have consistently reported a higher prevalence of radiographic knee osteoarthritis (knee ROA) in women than in men from middle age onwards [1]. Similarly, knee pain tends to be more frequently reported by women than by men [2,3]. One might expect there to be a corresponding excess of symptomatic knee OA (the combination of knee pain and ROA) among women. However, two recent studies challenge this. Using NHANES III data, Dillon and colleagues found no significant gender difference in the prevalence of symptomatic knee OA in adults aged 60 years and over. Yet women in this study had a higher prevalence of knee ROA and a higher prevalence of knee pain [2,4]. Another recent study conducted amongst symptomatic adults aged 50 years and over, drawn from the general population in North Staffordshire, England, found a significantly higher proportion of symptomatic men had knee ROA compared to symptomatic women. In this cohort, known as CAS(K) (the Clinical Assessment Study of the Knee), the finding was not explained by differences between male and female study participants in the distribution of several known risk factors such as age, body mass index (BMI), previous injury, or other clinical signs and symptoms [5]. We felt it was important to examine possible explanations for the excess of male symptomatic knee ROA in the CAS(K) cohort in order to determine if an artefact of study design was responsible, in which case important lessons for future study design could be learned and misinterpretation avoided.

In this article, we investigate four possible explanations for this excess of knee ROA in symptomatic males:

1. the effect of using comprehensive X-ray views to define knee ROA. Many previous studies have been limited to a single view of the tibiofemoral joint only, yet the inclusion of skyline and lateral X-ray views is likely to increase the overall proportion of cases as well as affect the compartmental distribution [6]. The difference in symptomatic knee ROA between the genders in CAS(K) may disappear when tibiofemoral joint osteoarthritis (TJOA) data from the PA view alone is assessed;

2. the effect of basing selection to the CAS(K) study on symptom status and a multiple-stage process. Most previous population studies have used a single-stage sampling technique to recruit both symptomatic and asymptomatic individuals. There may have been selective non-participation in research clinics by CAS(K) females;

3. the effect of occupation. Two of the three general practices involved in the study covered mining and farming communities, and there is a high level of scientific evidence for a relationship between occupational activity and knee OA [7]. CAS(K) men may have a higher level of occupational exposures associated with a risk of knee OA compared to women in the cohort;

4. the effect of pain arising from non-articular conditions, e.g. referred pain, peri-articular causes. Since CAS(K) participants were recruited on the basis of knee pain, if females had more non-articular sources of knee pain than males, women may have fewer radiographic changes associated with their knee pain than men.

## Methods

CAS(K) is a large prospective cohort study of knee pain and knee OA in symptomatic adults drawn from the general population in North Staffordshire, England. Approval for this study was granted by North Staffordshire Local Research Ethics Committee (project number 1430).

The detailed methodology of CAS(K) has been published previously [8]. Briefly, all patients aged 50 years and over registered with three general practices in North Staffordshire (covering a mix of urban and rural areas) were sent a two-stage postal survey. Approximately 98% of the UK population is registered with a GP [9], and therefore the GP register is regarded as being representative of the general population in the UK [10]. Responders who reported knee pain in the previous 12 months, and returned both questionnaires, were invited to attend a research assessment clinic consisting of clinical interview, physical examination, digital photography, plain X-ray, anthropometric measurement and brief self-complete questionnaire. Baseline data collection took place between August 2002 and September 2003.

6108 of 8984 adults returned the baseline Health Survey (adjusted response 70%), of whom 3106 reported knee pain in the previous 12 months [11]. Of these, 2226 consented to further contact and were mailed the Regional Pains Survey. 1949 responded (adjusted response 88%), of whom 819 attended the research assessment clinics.

### Radiography

Three radiographic views were obtained from each participant (weight-bearing posteroanterior (PA) semiflexed/metatarsophalangeal view; supine skyline; and supine lateral). A Kellgren and Lawrence (K & L) score was assigned to the PA and skyline views using the original written description, which included the presence of a 'definite' osteophyte for grade 2 [12]. A standard atlas was used to score superior and inferior osteophytes for the lateral view [13]. Posterior tibial osteophytes were scored on the same basis of severity as osteophytes in the lateral view. Any knee ROA was defined as a K & L score ≥2 on the PA view and/or a K & L score ≥2 on the skyline view and/or the presence of superior/inferior patella osteophytes on the lateral view and/or the presence of posterior tibial osteophytes on the lateral view. A single reader (RCD), blinded to all clinical and questionnaire data, scored all films. Intra- and inter-observer reliability were both very good (kappa 0.81–0.98 and 0.49–0.76 respectively) [6]. Information from all three radiographic views was used to define any knee ROA, severity of knee ROA (mild or moderate/severe), and TJOA and patellofemoral joint osteoarthritis (PJOA), as described previously [6,14].

### Occupational measures

Information on participants' occupation was obtained by asking about current employment status, current job title (if working) and last job title (if not working or retired). Occupational data were classified according to the Standard Occupational Classification (SOC2000) [15] using the current job title provided by participants, or the last job title if no current job title was given. The National Statistics Socio-economic Classification (NS-SEC) [16] was derived from SOC2000 occupational unit group and employment status.

### Non-articular conditions

Information from the clinical interview, physical examination and self-complete questionnaire was used to identify the presence of the following five non-articular conditions that could cause pain around the knee: full leg pain, widespread pain, low back pain with referred leg pain, possible hip arthritis and possible bursitides (pes anserine, prepatellar, infrapatellar) [17].

### Statistical analysis

Analysis was confined to baseline data from participants for whom we had complete X-ray data for their 'index knee' (defined by each participant as the most problematic knee). In the small number of cases where both knees were equally problematic, the index knee was randomly chosen. Exclusions were total knee replacement in index knee, an existing diagnosis of inflammatory arthropathy in the medical records, spoilt/missing films, or no knee pain in the last 6 months.

Radiographic information was used to define sub-groups with TJOA only, PJOA only, and OA in both compartments (i.e. combined TJOA/PJOA). For hypothesis 1 and 2a, we also defined a sub-group in which TJOA was present using only the PA view (i.e. the data from TJOA only added to that from combined TJOA/PJOA) so that comparisons could be made to previous studies. To further address hypothesis 2a, we took the same group of participants with complete index knee X-ray data, as described above, and within this defined (i) a sub-group with bilateral knee pain, which we further stratified for the presence of bilateral knee ROA and (ii) a sub-group with unilateral index knee pain only, which we further stratified for the presence of unilateral ROA in the index knee only, and unilateral ROA in the non-index knee only. Analyses to address hypotheses 1 to 4 were performed with data presented as percentages separately for males and females and stratified by age (50–64 years, 65 years and over). When comparing across genders, estimates of interest are expressed as percentage differences with 95% confidence intervals (CI).

## Results

Of the 819 participants who attended the clinical assessment, 745 were eligible and had complete X-ray data (54.6% female; mean age 65.2 years, SD 8.6 years; mean BMI 29.6 kg/m^2^, SD 5.2 kg/m^2^).

### Pattern of excess symptomatic knee ROA in men

Symptomatic male participants showed a statistically higher occurrence of any knee ROA compared to symptomatic female participants, with a percentage difference of 13.9% in 50–64 year olds, and 15.3% in the 65 years and over age group (Table 1). This excess in 50–64 year olds was due to a higher occurrence of mild knee ROA (not moderate/severe OA), and isolated PJOA (not combined TJOA/PJOA) in men. In 65 years and over, the excess in men was seen for both levels of OA severity and in combined TJOA/PJOA.

**Table 1:** Severity and compartmental distribution of symptomatic knee ROA, according to age and sex*

***Age 50–64 years***				
	Females (n = 200)	Males (n = 158)	% difference	95% CI

Any knee ROA (all 3 views; any compartment), n (%)	100 (50.0%)	101 (63.9%)	13.9%	3.6%, 23.8%
Severity of ROA (all 3 views; any compartment), n (%)				
Mild	56 (28.0%)	68 (43.0%)	15.0%	5.1%, 24.7%
Moderate/severe	44 (22.0%)	33 (20.9%)	-1.1%	-9.5%, 7.6%
Compartmental ROA, n (%)				
PJOA only	37 (18.5%)	55 (34.8%)	16.3%	7.1%, 25.4%
TJOA only	10 (5.0%)	7 (4.4%)	-0.6%	-5.1%, 4.4%
Combined TJOA/PJOA	53 (26.5%)	39 (24.7%)	-1.8%	-10.7%, 7.4%

***Age 65+ years***				

	Females (n = 207)	Males (n = 180)	% difference	95% CI

Any knee ROA (all 3 views; any compartment), n (%)	150 (72.5%)	158 (87.8%)	15.3%	7.4%, 22.9%
Severity of ROA (all 3 views; any compartment), n (%)				
Mild	42 (20.3%)	51 (28.3%)	8.0%	-0.5%, 16.6%
Moderate/severe	108 (52.2%)	107 (59.4%)	7.3%	-2.6%, 16.9%
Compartmental ROA, n (%)				
PJOA only	43 (20.8%)	43 (23.9%)	3.1%	-5.1%, 11.5%
TJOA only	10 (4.8%)	3 (1.7%)	-3.2%	-7.2%, 0.6%
Combined TJOA/PJOA	97 (46.9%)	112 (62.2%)	15.4%	5.4%, 24.9%

*Figures are based on 745 participants in CAS(K) – exclusions are total knee replacement in index knee, inflammatory arthropathy, spoilt/missing films, or no knee pain days in last 6 months.

ROA = Radiographic osteoarthritis; PJOA = Patellofemoral joint osteoarthritis; TJOA = Tibiofemoral joint osteoarthritis.

### Hypothesis 1. X-ray views taken

Percentages of participants with TJOA were calculated based solely on the data from the PA view. This analysis showed there was no gender difference for 50–64 year olds (M: 29.1%, F: 31.5%) but there was still an excess of males with PA view TJOA (63.9%), compared with females (51.7%), in the 65 years and over age group.

### Hypothesis 2a. Selection based on symptom status

In the Framingham cohort of individuals aged 63 years and over, the proportion of individuals with knee ROA (PA view only) amongst those with symptoms on most days in the past month was 43% of males and 73% of females [18]. Applying the same definition to the CAS(K) cohort aged 65 years and over, the proportion of individuals with knee ROA (PA view only) amongst those with symptoms on most days in the past month was still greater in males (61%) compared to females (52%).

Further analysis of CAS(K) data showed that the proportion of those with knee symptoms that were bilateral was similar for men and women within each age stratum (Table 2). However, amongst those with bilateral knee symptoms, there was still a significantly higher proportion of males than females with bilateral knee ROA in the 65 and over age group (percentage difference 12.2%; 95% CI: 2.0%, 21.8%). In those with unilateral (index) knee symptoms only, there was a significant excess of male index knee ROA in both age strata, although the numbers for analysis were small (50–64 years: percentage difference 32.0%; 95% CI: 12.4%, 48.2%; 65+ years: percentage difference 28.7%; 95% CI: 9.9%, 44.9%). Furthermore, in those with index knee pain, there was still a significant excess male non-index knee ROA in those aged 65 and over (percentage difference 25.7%; 95% CI: 4.4%, 44.0%).

**Table 2:** Proportions of males and females with bilateral, and unilateral, knee pain, stratified by knee ROA

	Females		Males	
		
Age (years)	50–64	65+	50–64	65+
Bilateral knee pain	148/200 (74%)	163/207 (79%)	111/158 (70%)	139/180 (77%)
+ Bilateral knee ROA	65/148 (44%)	108/163 (66%)	55/111 (50%)	109/139 (78%)
Unilateral knee pain (index knee)				
+ Unilateral ROA (index knee)*	21/52 (40%)	26/44 (59%)	34/47 (72%)	36/41 (88%)
+ Unilateral ROA (non-index knee)**	22/52 (42%)	19/40 (48%)	23/46 (50%)	30/41 (73%)

*n = 184 (index knee exclusions are total knee replacement, inflammatory arthropathy, spoilt/missing films, or no knee pain days in last 6 months).

**n = 179 (non-index knee exclusions are total knee replacement, amputation, spoilt/missing film).

ROA = Radiographic osteoarthritis

### Hypothesis 2b. Selection bias

There were 3106 participants who reported knee pain in the last 12 months and hence were eligible for inclusion in the clinical assessment. 819 symptomatic participants (26%) in this group completed the clinical assessment. There was some difference in participation rate in the research clinics by gender and age. Of the 3106, participation in clinics in the younger age group was similar across genders (29% in females and 30% in males), whilst in the 65+ age group females were less likely to attend the clinical assessment than males (17% vs. 29%). A previously published comparison of the research clinic attendees to all the respondents who reported knee pain in the last 12 months (n = 3106) showed selective non-participation of persons aged 80 years and over, females, not married/cohabiting, those with lower educational attainment or from lower socioeconomic groups (less likely to consent to further contact and to attend research clinic), those in employment, those experiencing anxiety or depression, or those reporting only a brief episode of knee pain within the previous year (less likely to attend research clinic) [11].

Examination of selection with respect to age, gender and NS-SEC occupational class is presented in Table 3. Of the participants in the four age/gender groups eligible to attend clinic, symptomatic females aged 65 years and over had the lowest level of participation. Further stratification by NS-SEC occupational class at the same selection points showed that in those aged 50–64 years eligible to attend clinic, there was a higher proportion of males than females in managerial and professional occupations who attended clinic, but a higher proportion of females than males in the other two occupational classes. In those aged 65 years and over eligible to attend clinic, there was a higher proportion of males than females in all three occupational classes who attended clinic.

**Table 3:** Proportions of males and females at two selection points, stratified by age and NS-SEC class

	Females		Males	
		
	Reported knee pain in last 12 months	Attended research clinic	Reported knee pain in last 12 months	Attended research clinic
	(n = 1832)	(n = 440)	(n = 1274)	(n = 379)
Age (years)				
50–64	751	209 (27.8%)	630	167 (26.5%)
65+	1081	231 (21.4%)	644	212 (32.9%)

NS-SEC class for age 50–64 years				
Managerial & professional occupations	107	36 (33.6%)	120	56 (46.7%)
Intermediate occupations	109	40 (36.7%)	93	25 (26.9%)
Routine & manual occupations	478	121 (25.3%)	384	79 (20.6%)

NS-SEC class for age 65+ years				
Managerial & professional occupations	91	37 (40.7%)	116	64 (55.2%)
Intermediate occupations	171	56 (32.7%)	103	39 (37.9%)
Routine & manual occupations	662	123 (18.6%)	380	97 (25.5%)

NS-SEC = National Statistics Socio-economic Classification

### Hypothesis 3. Occupation

The profile of NS-SEC classes differed according to gender (Table 4). A larger proportion of symptomatic women than men reported working in intermediate, and routine and manual, occupations. However, within each NS-SEC class there was a higher percentage of symptomatic males with knee ROA than females. The biggest percentage difference between the genders (18.3%), and the only value to reach statistical significance (due to insufficient numbers in the other two strata), was found in the routine and manual occupations class. Stratification of occupational data into 50–64 years old and 65 years and over age groups did not alter the gender difference (data not shown).

**Table 4:** Proportions of males and females with symptomatic knee ROA, stratified by NS-SEC class*

NS-SEC class	Females with knee ROA (%)	Total females in NS-SEC class	Males with knee ROA (%)	Total males in NS-SEC class	% difference between females & males with knee ROA	95% CI
Managerial & professional occupations	39 (56.5%)	69	71 (68.9%)	103	12.4%	-2.1%, 26.7%
Intermediate occupations	58 (68.2%)	85	49 (81.7%)	60	13.4%	-1.2%, 26.5%
Routine & manual occupations	139 (61.0%)	228	126 (79.2%)	159	18.3%	9.0%, 26.8%

Total	236	382	246	322		

*Figures are based on 745 participants in CAS(K) – exclusions are total knee replacement in index knee, inflammatory arthropathy, spoilt/missing films, or no knee pain days in last 6 months.

NS-SEC = National Statistics Socio-economic Classification; ROA = Radiographic osteoarthritis

### Hypothesis 4. Non-articular conditions

Overall, 37% of participants had one or more non-articular conditions (Table 5). In those aged 50–64 years, the proportions of symptomatic males and females with at least one non-articular condition was similar (percentage difference 3%; 95% CI: -7%, 13%). This was also seen when the data was stratified by the presence of knee ROA, although numbers for analysis were small. However, in the 65 years and over age group, there was a statistically higher proportion of females than males with at least one non-articular condition (percentage difference 10%; 95% CI: 4%, 19%). This difference between the genders was seen in those with and without knee ROA (Table 5).

**Table 5:** Proportions of symptomatic males and females with ≥1 non-articular condition, stratified by knee ROA

	Females		Males	
		
Age (years)	50–64 (n = 200)	65+ (n = 207)	50–64 (n = 158)	65+ (n = 180)
≥1 non-articular condition	78/200 (39%)	84/207 (41%)	57/158 (36%)	55/180 (31%)
Any knee ROA	37/100 (37%)	61/150 (41%)	37/101 (37%)	49/158 (31%)
No knee ROA	41/100 (41%)	23/57 (40%)	20/57 (35%)	6/22 (27%)

ROA = Radiographic osteoarthritis

## Discussion

Our study found an apparently higher occurrence of knee ROA among symptomatic men in North Staffordshire compared to symptomatic women. In middle age, this reflected a higher occurrence in men of mild OA, particularly isolated patellofemoral joint radiographic OA. Above retirement age, the difference included more severe disease and the involvement of both the patellofemoral and tibiofemoral joints.

We investigated four possible reasons for the gender difference. If we ignored cases of knee ROA identified from skyline and lateral views, the gender difference did disappear in 50–64 year olds. However, in the older age group, there was still a clear excess of tibiofemoral joint radiographic OA in men. Therefore, our choice of comprehensive X-ray views does not fully explain our findings. In fact, the use of all three views is a strength of our study. A pattern of progression from isolated patellofemoral joint disease to more severe, multiple compartment disease is implied in our findings and should be investigated in longitudinal analyses. When we examined the age structure of the participants in the older age group in more detail, we found a similar distribution of males and females in those aged 65–79 years and in those over 80, confirming that age imbalance in the 65+ age group was not a possible explanation for our findings.

Unlike previous radiographic studies of the knee that have included both symptomatic and asymptomatic individuals [18] our cohort consisted entirely of symptomatic individuals. Together with differences between studies in the definitions of "symptomatic", this means that direct comparisons are difficult. Yet we were able to identify individuals with "symptoms on most days in the previous month", a definition used previously. When we selected comparable subgroups of symptomatic individuals using this definition, the occurrence of knee ROA in our cohort was still higher in men, the opposite direction to the Framingham cohort [18]. The occurrence of knee ROA in North Staffordshire men was higher than in Framingham (61% vs. 43%), whilst the occurrence in women was lower than in Framingham (52% vs. 73%). This would suggest not simply an excess risk among North Staffordshire men but also a reduced risk (or higher levels of protective factors) among North Staffordshire women. Nor was the gender difference restricted to one knee. Even though there were similar proportions of men and women with bilateral knee symptoms in each age group in the CAS(K) cohort, there was still an excess of male compared to female bilateral knee ROA amongst those with bilateral knee symptoms in the older age group. Since the gender difference also persisted when we compared male asymptomatic knees with female asymptomatic knees, we do not believe that selection to the study based on symptom status was responsible for the gender pattern.

Factors associated with selective non-participation in the CAS(K) research clinics have been identified and reported and, as only 26% of the total observed potentially eligible population were recruited into the clinic stage, selection bias, particularly selective non-participation of symptomatic older women in the research clinics, must remain a possible explanation [11]. Indeed, previous studies have suggested that the group with the highest prevalence of knee ROA is the oldest women [4] and as this group is under-represented in the CAS(K) cohort, the direction of our selection bias would be to under-estimate the prevalence of knee ROA among the women thereby leading to an apparently higher prevalence in men in this age group. However, age- and gender-stratified mean WOMAC scores in clinic attendees were almost identical to those found in a single-stage postal survey with high response in North Staffordshire conducted just two years prior to this study [19]. Furthermore, of the participants eligible to attend clinic, the selective non-participation of routine and manual workers occurred in both genders and age groups. To approach the Framingham results, selection bias would need to result in a selective non-participation of symptomatic men *without *knee ROA, with or without selective non-participation of symptomatic women *with *knee ROA. The absence of X-ray data on non-participants prevents us from exploring this further. However, it is possible to envisage a process whereby attending research clinics was inconvenient for men at low risk of knee ROA (e.g. younger, healthy workers, mild symptoms, normal BMI) and also for women at high risk of knee ROA (e.g. older, living alone, more severe symptoms, limited mobility), where risk relates to both knees.

An alternative explanation is that there was a genuinely higher susceptibility or exposure among men in the CAS(K) cohort compared to women, for which occupational exposures may have been responsible. However, occupation did not appear to explain our findings. The excess of knee ROA in men compared to women was essentially the same for professional and managerial workers as for routine and manual occupations. There are, however, several weaknesses in this analysis that prevent the role of occupational exposures being ruled out. Firstly, although some effect would still be expected to be seen, the classification of occupation was relatively crude (owing to insufficient numbers for the statistical analysis of more detailed categorisation), not specifically based on the physical exposures likely to confer risk of knee OA (job title rather than job activity, e.g. repetitive knee bending), and not necessarily resulting in a fair comparison between men and women. Electricians/electrical fitters, labourers in process & plant operations, van drivers, coal mine operatives, glass & ceramics makers decorators & finishers, HGV drivers, stock control clerks, auto electricians, labourers in building & woodworking trades, metal working machine operatives, and goods handling & storage occupations accounted for 51% of men in routine or manual occupations. Of these, 86% had knee ROA. These are occupations with a high likelihood of knee bending activity [20,21]. The jobs held among women in routine and manual occupations were nearly all different from those held by men, the most common being sales & retail assistants, elementary office occupations, care assistants & home carers, labourers in process & plant operations, cleaners & domestics, chefs and cooks, kitchen & catering assistants, glass & ceramics makers decorators & finishers, school mid-day assistants and textile process operatives. These occupations accounted for 72% of women in routine or manual occupations, of which 60% had knee ROA. Secondly, occupational classification was based on current or last job title only. If the observed pattern of knee ROA is linked to occupation, we would need to identify the job held for the longest time (main job) as a better proxy of the cumulative effect of lifetime occupational exposures (the period of exposure for this cohort would date back to approximately 1924 for some individuals). A previous study in North Staffordshire found that main job was also the current job in only 25% of adults aged 50 years and over [Lacey, unpublished data]. Hence, current occupation may well under-estimate and misclassify lifetime occupational exposures. For example, men may have had more continuous employment than women, since in a previous North Staffordshire survey, men had worked for a mean of 28 years in their main job, compared to 20 years for women, in those aged 50 and over [Lacey, unpublished data]. Furthermore, if men were more likely to move from manual to professional or managerial work this may explain why knee ROA was still seen more commonly in men whose current or last job was in professional or managerial positions than in women.

The final possibility we examined was that knee pain arising from a location quite distinct from that associated with radiographic findings was responsible for our findings. If women in the CAS(K) cohort had an increased prevalence of non-articular sources for pain compared to men, this might explain why women had relatively less symptomatic knee ROA than men. We found this to be the case in the older age group of our cohort. Although this could be a plausible explanation for the excess of symptomatic knee ROA among men in this study, the higher occurrence of non-articular conditions in women than men was seen equally in symptomatic participants with knee ROA as well as in those without knee ROA. Non-articular conditions, therefore, do not offer a clear alternative explanation of pain that could be attributed to symptomatic knee ROA.

Our study does have some limitations. Firstly, we did not collect data on sports and recreational activities, which are considered risk factors for knee OA [7], although the same recommendations conclude that the risk of OA associated with sport is less than that associated with a history of trauma and overweight [7]. Furthermore, we have previously found that a history of knee trauma did not explain the observed pattern of knee ROA in our study [5]. Indeed, the excess knee ROA in our study was due to PJOA in the younger men, which argues against a straightforward role for previous knee injury. Secondly, our participants may not be representative of the UK population as a whole although, in general, the age and gender distribution of the patients in the three study general practices was similar to that of the wider Staffordshire region for the same age group [22].

## Conclusion

Ultimately, the excess of knee ROA among symptomatic men in this sample from North Staffordshire seems unlikely to be attributable to the use of comprehensive X-ray views. In the absence of more detailed data on occupational exposures and non-articular conditions, selective non-participation bias seems the most likely explanation. This is likely to be a consequence of higher rates of structural problems in symptomatic men self-selected into the study compared to symptomatic women. The impact of bias associated with selective participation differs depending on the study design. An important lesson for the design of future observational studies that intend to make inferences about the general population is that there may be potential biases associated with differential self-selection in symptomatic populations.

## Competing interests

The authors declare that they have no competing interests.

## Authors' contributions

All authors participated in the design of the study and drafting the manuscript. All authors read and approved the final manuscript.

## Pre-publication history

The pre-publication history for this paper can be accessed here:


